# Climate change induces carbon loss of arable mineral soils in boreal conditions

**DOI:** 10.1111/gcb.16164

**Published:** 2022-04-01

**Authors:** Jaakko Heikkinen, Riikka Keskinen, Joel Kostensalo, Visa Nuutinen

**Affiliations:** ^1^ Natural Resources Institute Finland (Luke) Jokioinen Finland; ^2^ Natural Resources Institute Finland (Luke) Joensuu Finland

**Keywords:** Bayesian analysis, boreal, climate change, cropping system, soil carbon

## Abstract

One‐fourth of the global soil organic carbon (SOC) is stored in the boreal region, where climate change is predicted to be faster than the global average. Planetary warming is accelerated if climate change promotes SOC release into the atmosphere as carbon dioxide. However, the soil carbon‐climate feedbacks have been poorly confirmed by SOC measurements despite their importance on global climate. In this study, we used data collected as part of the Finnish arable soil monitoring program to study the influence of climate change, management practices, and historical land use on changes in SOC content using a Bayesian approach. Topsoil samples (*n* = 385) collected nationwide in 2009 and 2018 showed that SOC content has decreased at the rate of 0.35% year^−1^ on average. Based on the Bayesian modeling of our data, we can say with a certainty of 79%–91% that increase in summertime (May–Sep) temperature has resulted in SOC loss while increased precipitation has resulted in SOC loss with a certainty of 90%–97%. The exact percentages depend on the climate dataset used. Historical land use was found to influence the SOC content for decades after conversion to cropland. Former organic soils with a high SOC‐to‐fine‐fraction ratio were prone to high SOC loss. In fields with long cultivation history (>100 years), however, the SOC‐to‐fine‐fraction ratio had stabilized to approximately 0.03–0.04 and the changes in SOC content leveled off. Our results showed that, although arable SOC sequestration can be promoted by diversifying crop rotations and by cultivating perennial grasses, it is unlikely that improved management practices are sufficient to counterbalance the climate change‐induced SOC losses in boreal conditions. This underlines the importance of the reduction of greenhouse gas emissions to avoid the acceleration of planetary warming.

## Graphical abstract

By combining various geospatial databases with data of the national soil monitoring network, we explored the effects of climate change, soil properties, management practices, and historical land‐use on soil carbon content in boreal arable soils. One of our main findings is that increased summer temperature and precipitation due to climate change have resulted in soil carbon loss. This is concerning as it counteracts carbon sequestration by improved management and constitutes feedback that may exacerbate planetary warming.

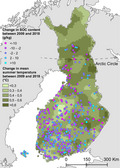

## 1 INTRODUCTION

The preservation of the soil organic carbon (SOC) pool is essential for both maintaining key soil functions, as well as mitigating climate change (Baveye et al., 2020; Stockmann et al., 2015). Soil organic carbon has an all‐embracing influence on soil fertility (pH, cation exchange capacity, nutrient reservoir), structure and stability (water and gas flow regulation, mechanical impedance, erodibility), biodiversity, and resilience (Obalum et al., 2017). Even small losses from the SOC pool may have considerable effects on the global climate since the organic C stored in the worlds' soils (ca. 1400–1600 Pg in the top 1 m layer) exceeds the amount of C in the atmosphere and plant biomass combined (Batjes, 2016; Scharlemann et al., 2014).

The size of the SOC pool depends on the balance between C inputs and C losses and is controlled by climate factors governing primary productivity and decomposition, in addition to soil geochemistry which affects C stabilization processes (Basile‐Doelsch et al., 2020; Doetterl et al., 2015; Kramer & Chadwick, 2018; Paul, 2016). Soil consists of SOC pools of varying residence times that can persist for up to millennia (Balesdent et al., 2018). Therefore, changes in SOC stocks may occur slowly (Smith, 2004), and observed SOC contents reflect not only the present management and climate but also the historical land use and past management (Stevens & Van Wesemael, 2008; Van Wesemael et al., 2010).

Globally, approximately one‐fourth of the SOC stock (0–30 cm) can be found in the boreal region (calculated based on the GSOCmap (FAO & ITPS, 2020), see also Batjes, 2016; Scharlemann et al., 2014) where temperature increase due to global warming is predicted to be higher than global average and rainfall is expected to increase (IPCC, 2021). This emphasizes the importance of northern soils in climate control. Warming and increasing soil moisture content accelerate decomposition and increase soil respiration, as they stimulate the microbial community (Lu et al., 2013; Pries et al., 2017), although extreme soil moisture has an inhibitory effect on respiration (Wickland & Neff, 2008). Simultaneously, more favorable agroclimatic conditions in northern regions are likely to enhance plant productivity and, consequently, soil C inputs (Churkina & Running, 1998; Trnka et al., 2011). It is, therefore, crucial to assess the balance of these contrasting processes considering the risk that climate change will be accelerated through SOC being released into the atmosphere as carbon dioxide (i.e., positive carbon cycle—climate feedback; see e.g., Bradford et al., 2016). Recently, the SOC cycle response and feedback to climate change have been studied using ecosystem modeling (Lugato et al., 2014; Riggers et al., 2021), field‐ and mesocosm‐based warming experiments (Melillo et al., 2017; Poeplau et al., 2017), and global databases of soil‐to‐atmosphere respiration (Bond‐Lamberty et al., 2018). Repeated soil surveys with large spatial coverage may enable the exploration of climatic impacts in natural state conditions using direct SOC measurements combined with data on climate, soil properties, and management. There are soil‐survey‐based studies indicating the increased risk for SOC losses as a result of a warming climate (Bellamy et al., 2005; Fantappiè et al., 2011; Heikkinen et al., 2013; Prietzel et al., 2016). However, the quantification of climatic impacts has proved difficult most likely due to high uncertainty related to the SOC measurements (Goidts et al., 2009; Smith et al., 2020).

Adopting improved agri‐environmental management strategies has been shown to have a high potential for increasing SOC levels (e.g., Minasny et al., 2017), though challenges have also been recognized (Baveye et al., 2020; Powlson et al., 2011). Increases in SOC stocks can be achieved by increasing C inputs (e.g., cover crops, organic amendments) and/or reducing decomposition and erosion losses through minimum soil disturbance and continuous soil cover. The effectiveness of these measures is site‐specific. In the context of European croplands, potential SOC sequestration rates have been estimated to range from 300 to 1900 kg C ha^−1^ year^−1^ when converting arable land to woodland or grassland and reach up to 700 kg C ha^−1^ year^−1^ when applying organic residues, zero‐ or reduced‐tillage or growing perennial or deep‐rooting crops (Freibauer et al., 2004). Recent studies have also shown that high plant diversity increases SOC storage by increasing primary production, belowground C inputs, and microbial activity converting the organic inputs to persistent compounds (Chen et al., 2018; Lange et al., 2015). Improved knowledge about the impact of these different management practices plays a key role in the effective mitigation of SOC loss.

The objective of this study was to quantify in decadal time scale the impact of climate change on SOC in boreal arable soils, taking simultaneously into account the roles of present management and historical land use. Furthermore, we investigated whether the possible loss of SOC due to climate change can be offset by adopting SOC enhancing management practices such as improved crop rotations. We used data from the Finnish arable soil monitoring network's mineral soil sites (OM < 20%) in investigating the effects of changing summertime temperature and precipitation and different cropping systems on topsoil (0–15 cm) SOC content. Since the changes in SOC stocks occur slowly (Smith, 2004), we also examined the role of historical land‐use and management on SOC changes with the aid of historical maps. The data were analyzed using advanced statistical methods to tackle challenges related to the inherently high variability of SOC measurements.

## 2 MATERIALS AND METHODS

### 2.1 Datasets

This study was based on four geospatial datasets: the national soil monitoring network for Finnish agricultural soils, the database of management and cultivated crop plants by the Finnish Food Authority, and two different climate grids: The E‐OBS climate grid (Cornes et al., 2018) provided by European Climate Assessment and Dataset, and the climate grid by Finnish Meteorological Institute (FMI).

**FIGURE 1:**
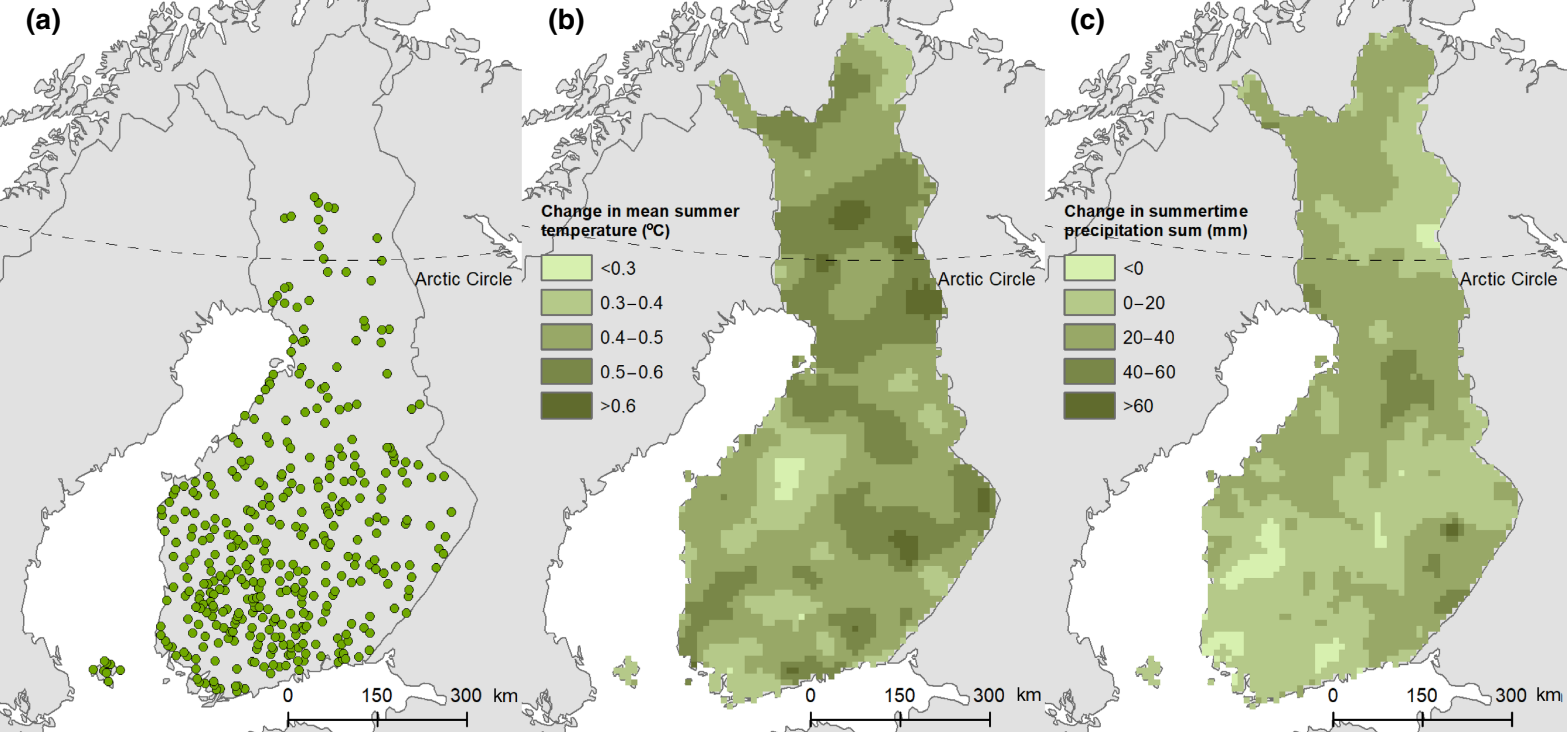
Location of sampling plots of the Finnish arable soil monitoring network utilized in the present study (*n* = 385) (a) and the change in summertime mean temperature (b) and precipitation sum (c) between 2009 and 2018 in 10 km × 10 km grid according to climate grid by Finnish Meteorological Institute

**FIGURE 2:**
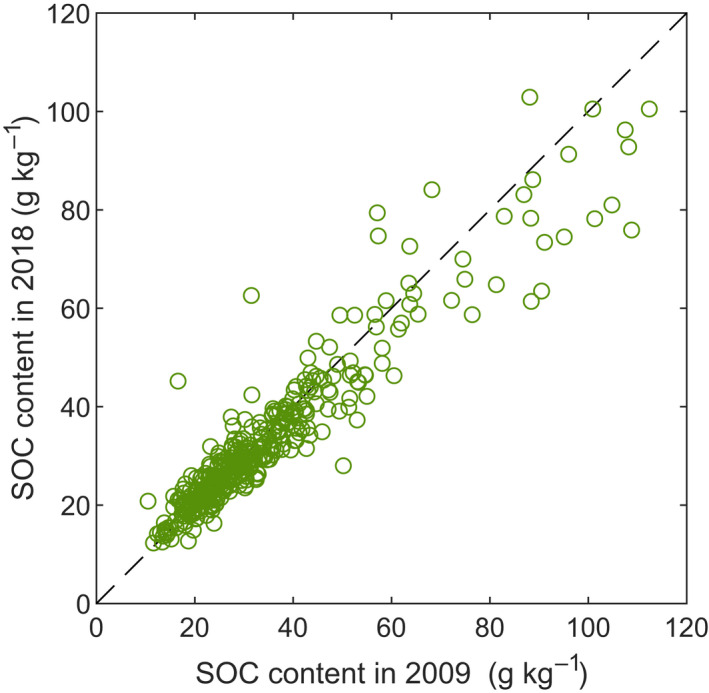
Measured SOC contents in 2009 and 2018 (*n* = 385). The dashed line represents the identity line with both values being equal

The soil monitoring network was established in 1974 and plots were resampled in 1987, 1998, 2009, and 2018. Sampling plots were set up all over Finland except for the most northern parts (Figure 1). This study was based on the SOC contents of soil samples collected in 2009 and 2018 (Figure 2) as the accurate GPS‐based location of the sampling plots was established in 2009. In 2018, the whole network consisted of 631 sampling plots. This study focused on the croplands as defined in the Finnish greenhouse gas inventory (Statistics Finland, 2021) based on the IPCC guidelines (IPCC, 2006; see also Heikkinen et al., 2013). After omitting organic soils (OM > 20%, *n* = 72), plots that were not actively cultivated (grasslands) or cultivation history was missing (*n* = 24), and new 150 plots established in 2018 and sampled only once, the total number of sampling plots included in this study was 385. The soil monitoring network has been described in detail by Heikkinen et al. (2013, 2021) and Keskinen et al. (2016).

**FIGURE 3:**
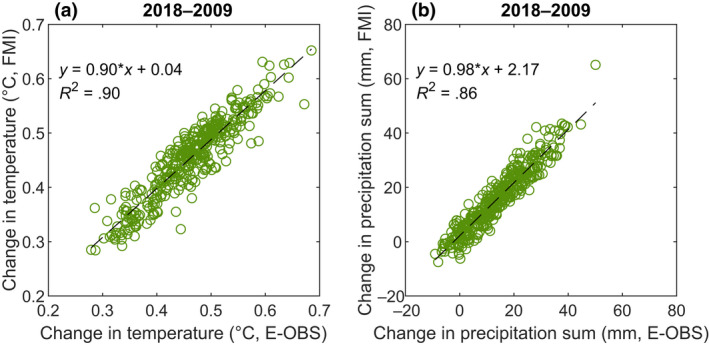
Comparison of change in summer (May–Sep) temperature (a) and precipitation sum (b) obtained from Finnish Meteorological Institute (FMI) and E‐OBS climate grids (*n* = 385)

Two sources were used to obtain summertime (May–Sep) weather data: the 0.1 degree (≈4–12 km) regular grid of Ensembles Observations dataset (E‐OBS) and the 1 km × 1 km monthly climate grid by the Finnish Meteorological Institute (FMI). Summertime weather was used as it is the most crucial period in Finland in respect of both decomposition of SOC and soil C inputs through biomass production. The rest of the year has a minor impact on SOC decomposition due to low topsoil temperatures and frost and snow cover during the winter months. In both datasets, the nearest grid point to the soil monitoring sampling plot was selected. On average, the two data sets are in good agreement with respect to temperature measurements, though a change of 1°C in the E‐OBS data set only predicts a change of 0.90°C in the FMI data set when a simple linear model is fitted (Figure 3). In the precipitation measurements, there is a statistically significant difference between the two data sets. In the FMI data, the mean change in summertime precipitation is 15.9 mm, while the mean change in precipitation is 14.1 mm in the E‐OBS data set. On the contrary, a 1 mm change in the E‐OBS data set predicts a 0.98 mm change in the FMI data set by a simple linear model (Figure 3).

The database by the Finnish Food Authority includes field parcel‐level information on cultivated crop species and farm type (plant production or livestock farm), with annual data available from 1995 onwards. Field parcels were identified using field‐specific identification codes determined based on the coordinates of the sampling plots.

Data were also analyzed using the measured SOC changes between 1998 and 2009 and the corresponding data for cultivation practices and climate change. The dataset has notably greater variation in SOC measurements than the 2009–2018 dataset due to the lack of accurate GPS‐based location of sampling plots and the results of the analysis are given separately in Supplement 1.

### 2.2 Sampling and analysis

Soil samples were obtained from the topmost 0–15 cm soil layer of 100 m^2^ (10 m × 10 m) sampling plots using a soil corer with a diameter of 2 cm. Representative composite samples of around 0.5 dm^3^ were mixed from 10 to 20 individual soil cores collected in each plot.

Samples were air‐dried, ground, and passed through a 2‐mm sieve. Soil organic C contents of the samples were determined by dry combustion method using LECO CN‐2000 analyzer (LECO). As Finnish agricultural soils are generally acidic, the samples were assumed to contain only organic carbon (Nelson & Sommers, 1996). Soil texture was determined using the sieve‐pipette method (Elonen, 1971).

### 2.3 Data classification

Sampling plots were classified according to the cropping system using the field parcel‐level data from 2009 to 2018 provided by the Finnish Food Authority. Records of cultivated crop plants were utilized to classify the crop rotation in four categories: diverse, annual, perennial, and rotation. The classification was based on the Shannon index (H) representing the diversity of the cropping system and the share of perennial grasses. Shannon index (H) was calculated using the equation

$$\text{Shannon Index}\left(H\right)=‐\sum_{i=1}^{s}p_{i}\;\ln\;p_{i},$$

where $p_{i}$ is the proportion (*n*/*N*) of the number of cultivation years of crop plant i (*n*) divided by the number of years in crop records (*N*, most cases *N* = 9), and *s* is the number of crop plants grouped into cereals, legumes, vegetables, oilseeds, grasses, green fallow, and others. If the Shannon index was higher than 0.8 the plot was classified as “Diverse.” Otherwise, classification was based on the prevalence of perennial grasses in the crop rotation. Plots growing annual or perennial crops more than 80% of the time were classified as “Annual” or “Perennial,” respectively. The remaining plots were categorized as “Rotation.” Furthermore, the sampling plots were classified into two groups depending on farm type: “Plant production” and “Livestock farm.”

The change in mean summertime temperature (May–Sept) between 2009 and 2018 was calculated for each sampling site as the difference in the mean summertime temperatures between the periods 1998–2018 and 1989–2009. The 20 years averaging period was used as it is long enough to smooth annual variations and the time periods do not overlap excessively. The change in precipitation sum was calculated similarly, although summertime (May–Sept) precipitation was summed up prior to averaging. The use of summertime climate data in the analysis is justified as in boreal conditions most of the decomposition of organic matter as well as the plant growth takes place in summer months. SOC‐to‐fine‐fraction ratio was calculated by dividing the SOC content in 2009 (g kg^−1^) with the content of the fine particle size fraction (<0.06 mm) in the soil (g kg^−1^).

**FIGURE 4:**
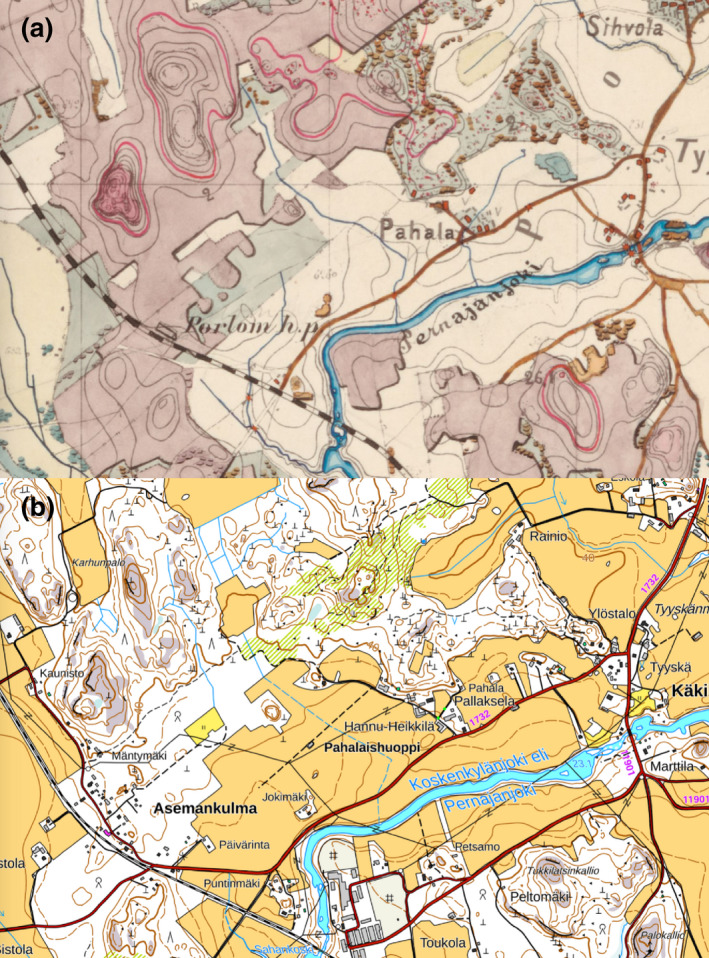
Exemplary map (www.vanhatkartat.fi) from 1874 (a), that was used to determine the historical land‐use of the sampling plots and the recent map from the same location (b)

Historical maps from the late 19th and early 20th centuries were used to identify the plots with over 100 years of cultivation (Figure 4). As the historical maps do not cover the whole country this information was, however, available only for 36% of the sampling plots. Former organic soils (having contained OM > 20%) were identified using the organic matter contents of the soil samples collected in previous sampling campaigns in 1987 and 1998.

### 2.4 Statistical analysis

Confidence intervals and *p*‐values for the mean change in SOC content over the time periods (2009–2018 and 1998–2009) were calculated using non‐parametric bootstrap (Efron & Tibshirani, 1994). A non‐parametric approach was chosen since the SOC change did not seem to follow any widely used distribution. The null hypothesis was that there would be no change in the average SOC content, and a two‐tailed alternative hypothesis where the SOC content has either increased or decreased on average was assumed. 100,000 bootstrap samples were used, and the simulation was implemented with the statistical software R (R Core Team, 2021).

The influence of crop rotation, farm type, changes in precipitation and temperature, and organic‐carbon‐to‐fine‐soil ratio (linked to the soil's ability to bind carbon) on changes in SOC content was examined using a Bayesian approach (Gelman et al., 2013). A model with these covariates and a measurement‐group‐specific random effect was constructed. The ratio of organic carbon to fine soil was log‐transformed since the range of values spanned several magnitudes. The distribution of the response variable (i.e., the change in SOC) was relatively symmetrical but had extremely thick tails, with excess kurtosis of roughly nine. This variation is most likely due to the high spatial variation of SOC at the field scale (Poeplau et al., 2022) and the limited accuracy in relocating exactly the same location. In order to account for the thick tales while simultaneously modeling a plausible data generating process, the response variable was assumed to follow a mixing distribution of two Gaussian distributions: one relatively narrow one for the successful relocations and a wider one for cases, where the measurement locations are spatially far enough apart so that the spatial variance in SOC levels is no longer adequately controlled for. However, even in the worst‐case scenario, the two measurement locations are going to differ only by some meters, so the climate and farming practice effects on SOC loss can be assumed to be on average the same. Thus, the expected values of the wide and narrow distributions were assumed to have equal expected values, i.e., have the same centering. We chose non‐informative prior distributions for all parameters apart from the variances of the two mixed Gaussian distributions, for which weakly informative priors based on graphical examination were chosen in order to ensure convergence (see Supplement 2 for the graphical argument). The model can be written as

$$\Delta SOC_{i}|\mu_{i},\sigma^{2}∼N\left(\mu_{i},\sigma_{i}^{2}\right),$$

$$\sigma_{i}|\pi ∼Cat\left(\sigma_{1},\sigma_{1}+\sigma_{2};\pi ,1‐\pi \right),$$

$$\sigma_{3}=\sigma_{1}+\sigma_{2},$$

$$\begin{array}{c} \mu_{i}∼\nu +\beta_{P}\Delta P_{i}+\beta_{T}\Delta T_{i}+\beta_{\mathrm{FT}}FT_{i}+\beta_{\mathrm{fine}}\log{\left(Org\;C/fine\;soil\right)}_{i}+\beta_{\mathrm{per}}Per_{i}\\ \;+\beta_{\mathrm{div}}Div_{i}+\beta_{\mathrm{rot}}Rot_{i}+u_{Group\left(i\right)}, \end{array}$$

$$u_{Group\left(i\right)}∼N\left(0,\sigma_{\mathrm{Group}}^{2}\right),$$

$$\beta_{P},\beta_{T},\beta_{\mathrm{FT}},\beta_{\mathrm{fine}},\beta_{\mathrm{per}},\beta_{\mathrm{div}},\beta_{\mathrm{rot}}∼N\left(0,10^{5}\right)$$

$$\pi ∼Dir\left(\left[1/\sqrt{2},1/\sqrt{2}\right]\right)$$

$$\sigma_{\mathrm{Group}}∼Unif\left(0.01,6\right)$$

$$\sigma_{1}∼Unif\left(0.01,6\right)$$

$$\sigma_{2}∼Unif\left(5,20\right),$$

where $\Delta SOC_{i}$ is the change in SOC for the plot i, $\Delta P_{i}$ is the change in precipitation, $\Delta T_{i}$ the change in summertime temperature, $FT_{i}$ is the farm‐type indicator which is 0 for plant farms and 1 for animal farms, $\log{\left(Org\;C/fine\;soil\right)}_{i}$ is the natural logarithm of the ratio of organic carbon and fine soil, and $Per_{i},Div_{i},Rot_{i}$ correspond to crop rotation types “perennial,” “diverse,” and “rotation,” respectively. The intercept ν corresponds to the expected change in SOC content for a plant‐type farm with annual crop rotation, no change in the climate variables, and an organic‐carbon‐to‐fine‐soil ratio OrgC/finesoil=1. The random effect $u_{Group\left(i\right)}$ accounts for possible variation between measurement groups and thus by proxy in the geographical area. The regression coefficients $\beta_{P},\beta_{T},\beta_{\mathrm{FT}},\beta_{\mathrm{fine}},\beta_{\mathrm{per}},\beta_{\mathrm{div}},\beta_{\mathrm{rot}}$ are given a prior distribution $N\left(0,10^{5}\right)$, which is within the region of interest close to a uniform distribution, while still being a true probability distribution and thus numerically implementable. The standard deviation $\sigma_{i}$ of the plot follows a categorical (two‐point) distribution where the value $\sigma_{1}$ has the probability π and $\sigma_{1}+\sigma_{2}$ the probability 1‐π. The parameter $\sigma_{3}$ is the standard deviation of the wider normal distribution.

The interpretation of model results is based on the posterior probabilities P(θ>0), where θ is the parameter of interest, e.g., $\beta_{P}$ which is the regression coefficient for the impact of changes in precipitation. Given that $P\left(\theta >0\right)>0.95$ can be seen as clear evidence that the parameter value is greater than zero, and $P\left(\theta >0\right)<0.05$ as clear evidence that the parameter value is smaller than 0. In the case of $\beta_{P},$ the first case indicates that increased precipitation is associated with a positive SOC change and in the latter case that an increase in precipitation is associated with SOC loss. Values above 0.9 or below 0.1 are reasonable indications since this means that one is more than 90% certain about whether a given factor is associated with an increase or a decrease in SOC. Therefore, in Bayesian analyses, it is customary to give the 80% probability intervals. For parameters such as standard deviations $P\left(\theta >0\right)=1,$ as these are positive numbers.

The models were fitted using five Markov chains with 300,000 iterations for each and a 50,000‐iteration‐burn‐in period. The fit was done using JAGS software (Just Another Gibbs Sampler; Plummer, 2013) via R. The convergence of the chains was graphically confirmed.

The quality of the fit was checked by calculating Bayesian *p*‐values for the minimum and maximum of the distribution (see e.g., Gelman et al., 2013 for the definition). The Bayesian *p*‐values are not analogous to *p*‐values of classical regression models but are instead quantities describing the quality of the fit like AIC or $R^{2}$ for a classical model. Furthermore, they should not be mistaken for the posterior probabilities P(θ>0) which describe the confidence in certain parameter values having a given sign and are analogous to classical *p*‐values. In order to avoid confusion, we give the posterior probabilities in percentages. A Bayesian *p*‐value tells how well the model reproduces a given feature of a distribution. A value between .025 and .975 can be seen as evidence that the model is in good agreement with the data. By investigating the minimum and maximum, we see whether the predicted tails of the distribution have the right length, or whether one of the tails is too thin or thick. We used a cross‐validation approach, where the data set was divided 10 times randomly into training data (80%) and test data (20%). After training the model with the training data, the probability of obtaining larger a maximum or a smaller minimum was calculated by drawing 100 random samples from the posterior predictive distribution of SOC change with the sample size equal to that of the test data.

## 3 RESULTS

### 3.1 Effect of climate change, cultivation practices, and soil properties on SOC content

The nationwide mean topsoil SOC content of cultivated mineral soils decreased from 34.1 g kg^−1^ in 2009 to 33.0 g kg^−1^ in 2018. Average annual SOC content change was −0.12 g kg^−1^ year^−1^ (95% C.I. (−0.19 g kg^−1^ year^−1^, −0.05 g kg^−1^ year^−1^), *p* < .001), which corresponds to a relative change of approximately −0.35%. The Bayesian model showed that climate change, cultivation practices, and soil properties all contributed to the change in SOC content (Tables 1 and 2).

**TABLE 1:** Effect of climate change, management practices, and SOC‐to‐fine‐fraction ratio on SOC content change $(E\left(\theta |y\right)$) using E‐OBS climate data, 80% (equally tailed) probability interval, and probability for positive effect (P(θ > 0)). The approximate effect on SOC stock change was calculated as $E\left(\theta |y\right)$/34.09 × 54, where 34.09 g/kg is the average SOC content in 2009 and 54 t C ha is the average nationwide SOC stock in 0–15 cm soil layer (Heikkinen et al., 2013). The climatic impacts on SOC stock changes (*) were calculated using the average increase in temperature (0.46°C) and precipitation sum (14 mm) between 2009 and 2018 taken from E‐OBS climate data

		Effect on SOC content change $E\left(\theta |y\right)$(g kg^−1^ year^−1^)	80%P.I. (g kg^−1^ year^−1^)	Probability for positive effect P(θ>0) (%)	Approximate effect on SOC stock change (kg C ha^−1^ year^−1^)
Climate change					
1 mm increase in precipitation sum	$\beta_{\text{change P}}$	−0.003	(−0.006, 0.0000)	10	−67*
$1^{\circ }$C increase in temperature	$\beta_{\text{change T}}$	−0.404	(−0.796, −0.010)	9	−296*
Farm type vs. “plant production”					
Livestock	$\beta_{\text{animal}}$	0.011	(−0.043, 0.066)	60	+17
Cropping system versus ‘annual’					
Diverse	$\beta_{\text{diverse}}$	0.081	(0.009, 0.152)	93	+128
Perennial	$\beta_{\text{perennial}}$	0.152	(0.068, 0.236)	98.7	+241
Rotation	$\beta_{\text{rotation}}$	0.137	(0.069, 0.204)	99.5	+217
SOC‐to‐fine‐fraction ratio (centered to mean)	$\beta_{\text{log}\left(\text{OrgC}/\text{fine}\right)}$	−0.193	(−0.234, −0.153)	<0.1	
Other parameters					
Constant	μ	0.048	(−0.144, 0.238)	63	+76
Narrow distribution fraction	π	0.734	(0.670, 0.799)	100	
Wide distribution fraction	1‐π	0.266	(0.201, 0.330)	100	
SD of narrow distribution	$\sigma_{1}$	0.267	(0.232, 0.304)	100	
SD of wide distribution	$\sigma_{3}$	1.178	(1.022, 1.333)	100	
Measurement group effect	$\sigma_{\text{group}}$	0.112	(0.062, 0.167)	100	

**TABLE 2:** Effect of climate change, management practices, and SOC‐to‐fine‐fraction ratio on SOC content change $(E\left(\theta |y\right)$) using FMI climate data, 80% (equally tailed) probability interval, and probability for positive effect (P(θ > 0)). The approximate effect on SOC stock change was calculated as $E\left(\theta |y\right)$/34.09 × 54, where 34.09 g/kg is the average SOC content in 2009 and 54 t C ha is the average nationwide SOC stock in 0–15 cm soil layer (Heikkinen et al., 2013). The climatic impacts on SOC stock changes (*) were calculated using the average increase in temperature (0.45°C) and precipitation sum (16 mm) between 2009 and 2018 taken from FMI climate data

		Effect on SOC content change $E\left(\theta |y\right)$(g kg^−1^ year^−1^)	80%P.I. (g kg^−1^ year^−1^)	Probability for positive effect P(θ > 0) (%)	Approximate effect on SOC stock change (kg C ha^−1^ year^−1^)
Climate change					
1 mm increase in precipitation sum	$\beta_{\text{change P}}$	−0.004	(−0.007, −0.001)	2.6	−101*
$1^{\circ }$C increase in temperature	$\beta_{\text{change T}}$	−0.244	(−0.644, 0.156)	21	−176*
Farm type versus “plant production”					
Livestock	$\beta_{\text{animal}}$	0.011	(−0.041, 0.067)	62	+17
Cropping system versus ‘annual’					
Diverse	$\beta_{\text{diverse}}$	0.094	(0.022, 0.166)	95	+149
Perennial	$\beta_{\text{perennial}}$	0.150	(0.068, 0.232)	99.9	+238
Rotation	$\beta_{\text{rotation}}$	0.136	(0.068, 0.202)	99.5	+215
SOC‐to‐fine‐fraction ratio (centered to mean)	$\beta_{\text{log}\left(\text{OrgC}/\text{fine}\right)}$	−0.194	(−0.234, −0.154)	<0.1	
Other parameters					
Constant	μ	−0.003	(−0.187, 0.180)	49	−5
Narrow distribution fraction	π	0.731	(0.670, 0.792)	100	
Wide distribution fraction	1‐π	0.269	(0.208, 0.330)	100	
SD of narrow distribution	$\sigma_{1}$	0.263	(0.231, 0.298)	100	
SD of wide distribution	$\sigma_{3}$	1.167	(1.033, 1.322)	100	
Measurement group effect	$\sigma_{\text{group}}$	0.112	(0.064, 0.164)	100	

Both E‐OBS and FMI climate data indicated that the increase in summertime (May–Sep) temperature and precipitation sum over the study period resulted in SOC loss. However, when E‐OBS data were used, the association between the SOC change and the change in temperature was stronger than in using the FMI data, whereas with the FMI data summertime precipitation sum had a more pronounced effect on the SOC content change. Using E‐OBS data the probabilities that increase in temperature and precipitation result in SOC loss were 91% and 90%, respectively (Table 1). Corresponding probabilities with FMI climate data were 79% and 97% (Table 2). In both climate datasets, temperature increase had a more pronounced impact on approximate SOC stock change than the increase in precipitation (Tables 1 and 2).

Effects of cropping systems on SOC were nearly identical regardless of the climatic data used (Tables 1 and 2). With respect to the accumulation of C into the soil, the perennial dominated, and the annual‐grass rotations were the most beneficial cropping systems followed by the diverse cropping system. The greatest risk for SOC losses was associated with cropping systems dominated by annual plants. In comparison to annual‐dominated rotation, the rate of SOC accumulation was 0.15, 0.14, and 0.08 g/kg C year^−1^ for perennial, crop rotation, and diverse cropping system, respectively (Table 1). With average SOC stock of 54 Mg C ha^−1^ (Heikkinen et al., 2013), this corresponds to an increase in SOC stock of about 241, 217, and 128 kg C ha^−1^ year^−1^. With respect to farm type, there was no clear difference in SOC balance between livestock and plant production farms (Tables 1 and 2).

Change in SOC content was especially strongly associated with the initial SOC‐to‐fine‐fraction ratio indicating the protective capacity of fine‐grained mineral particles in SOC stabilization processes. The higher the initial SOC‐to‐fine‐fraction ratio, the greater the SOC loss tended to be (Tables 1 and 2). Changes in SOC pools are slow and thus the SOC‐to‐fine‐fraction ratio can be influenced by historical land‐use of past decades. This is assessed more in‐depth in the next section “Historical land‐use and the change in SOC content.”

The data were also analyzed using the measured SOC changes between 1998 and 2009 and the corresponding data for cultivation practices and climate change (Supplement 1). With respect to the SOC‐to‐fine‐fraction ratio and cropping system, the results were generally parallel with the results of 2009–2018 data. However, due to considerably greater variation in SOC measurements (due to lack of GPS‐based locations) the signals related to the impact of climate change on SOC content remained weaker and were not detectable in the 1998–2009 data.

The obtained Bayesian *p*‐values for the four data sets (1998–2009 and 2009–2018, FMI and E‐OBS climate data) were between .27 and .71 indicating that the model can very accurately reproduce the observed distribution of SOC change.

### 3.2 Historical land‐use and the change in SOC content

As the change in SOC content was especially strongly associated with the SOC‐to‐fine‐fraction ratio (Tables 1 and 2), it deserves a closer look with respect to past land‐use of the sampling plots. This was examined using the historical maps and the results of the previous sampling campaigns of the soil monitoring network conducted in 1987 and 1998.

**FIGURE 5:**
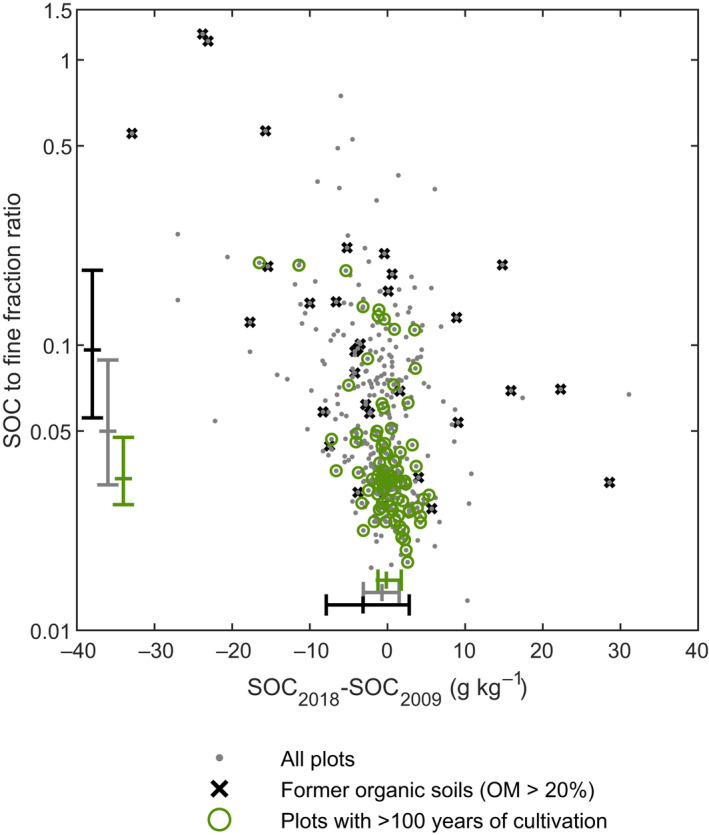
Relationship between the change in SOC content and the ratio of SOC to fine fraction contents. Former organic soils (OM > 20%) were identified based on the organic matter contents of the soil samples collected in 1987 and 1998. Plots with >100 years of cultivation were identified using the historical maps from the late 19th or early 20th centuries. Historical maps were not available for the whole country and thus “all plots”—category also includes those sampling plots of which land‐use history is not known. The range bars denote the interquartile range and median of the observed values

In comparison to all observations included in the study, the plots with more than 100 years long cultivation history tended to have lower SOC‐to‐fine‐fraction ratios (0.03(Q_1_)–0.05(Q_3_)) and consequently more modest change in SOC contents. Scatter in the data was also smaller as shown in interquartile ranges of the observed values in Figure 5.

Former organic soils (OM > 20%) were characterized by having more scatter both in SOC contents and in SOC‐to‐fine‐fraction ratios (Figure 5). Furthermore, SOC‐to‐fine‐fraction ratios of those plots were higher (0.06(Q_1_)–0.18(Q_3_)), and they tended to lose SOC at a notably higher rate than all plots on average. Median values for SOC‐to‐fine‐fraction contents were 0.10, 0.05, and 0.03 for plots with former organic soils, all observations, and plots with >100‐year cultivation history, respectively. Correspondingly, median values for the change in SOC contents between 2009 and 2018 were −3.2, −0.7, and −0.2 g kg^−1^.

## 4 DISCUSSION

### 4.1 Change in climate results in SOC loss

In Finland, the range of mean summer temperature increase in 1998–2018 has varied between 0.2–0.7°C in comparison to 1989–2009 (Figure 1). The lengthening of the active growing season due to climate warming in northern regions has been thought to increase the soil productivity (Myneni et al., 1997) and hence the C input to soil. However, in agricultural land, the soil C inputs are largely driven by anthropogenic factors and there is mounting evidence that the productivity of European croplands also in the boreal region has stagnated rather than being increased (Peltonen‐Sainio et al., 2015; Ray et al., 2012; Wiesmeier et al., 2015). The observed association between SOC loss and increase in temperature and precipitation in the present study indicates that soil C inputs have not increased sufficiently to counterbalance the losses due to more favorable conditions for decomposition. The results are concerning as it is predicted that in northern Europe the summer (May–Sept) temperature will increase by about 1.5–3°C from 1981–2010 baseline level to 2041–2060 depending on the climate scenario used (Gutiérrez et al., 2021). Correspondingly, precipitation is predicted to increase up to 10% (Gutiérrez et al., 2021).

The results of this study are in line with nearly two decades ago published model‐based study by Smith et al. (2005). Their study showed that climate change is the key driver of SOC change in European croplands and in the northern latitudes climate change results in SOC loss. Likewise, the model‐based studies by Riggers et al. (2021) and Stergiadi et al. (2016) suggested that with projected climate the cropland SOC stocks will decline in northwestern Europe whereas in southern Europe climate change might lead to the accumulation of SOC (Álvaro‐Fuentes et al., 2012). So far, the soil carbon‐climate feedbacks have been poorly confirmed by direct SOC measurements. Given that high latitude and altitude regions have the highest SOC stocks and among the fastest expected changes in climate, it does not come as a surprise that the evidence about the influence of climate change on SOC begins to emerge first from these regions (Bellamy et al., 2005; Prietzel et al., 2016; present study). The importance of high latitude regions in the global carbon cycle is further underlined by findings showing that soils with high SOC stock and in cold climatic regions are the most sensitive to warming (Crowther et al., 2016; Karhu et al., 2014; Koven et al., 2017).

The estimated effects on SOC stock (Tables 1 and 2) indicate that the change in summertime temperature has a more profound effect on the SOC content than the change in the precipitation. This is understandable as most of the boreal region falls into the moist climatic region where annual rainfall exceeds the potential evapotranspiration (see e.g., climate classification of IPPC (2006)). Decomposition of SOC in the boreal region is thus not generally limited by soil moisture availability and especially in autumn and spring after melting of the snow even mineral soils can be temporarily waterlogged. As a considerable part of the decomposition occurs during the few warmest summer months (decomposition rate increases exponentially with temperature), the seasonal and within‐season variation in rainfall pattern is, however, critical with respect to the overall effect of precipitation on SOC changes.

In other Nordic countries, in Denmark (Taghizadeh‐Toosi et al., 2014) and southeast Norway (Riley & Bakkegard, 2006), the climate change was postulated to be one possible reason for the observed decrease in SOC, along with contributions from land drainage and changes in crop rotation. In contrast, the SOC has been reported to increase in agricultural soils of Sweden and the change was mainly attributed to the expansion of ley area (Poeplau et al., 2015) indicating the importance of management practices on SOC sequestration.

### 4.2 Management practices and SOC

The observed ranking of cropping systems regarding SOC accrual (perennial > rotation > diverse > annual) is consistent with the current understanding of the effects of cropping systems on SOC. An increase in the share of perennial crops in the rotation stands out as the most beneficial management due to an increase in C input and decrease of tillage frequency (Deiss et al., 2021; King et al., 2020). Besides of extended period of biomass production, the input of root C, which is stabilized in the soil more efficiently than the aboveground plant residues, is emphasized in perennial systems (Anderson‐Teixeira et al., 2013; Kätterer et al., 2011; Rasse et al., 2005; Sokol et al., 2019). In Nordic conditions, soil C stocks increased at the rate of 0.4 t C ha^−1^ year^−1^ after conversion of arable land to grassland in Sweden (Kätterer et al., 2008) while Christensen et al. (2009) reported as high as 1.1 t C ha^−1^ year^−1^ accumulation of SOC during grass ley phase in Denmark. Likewise, Bolinder et al. (2010) showed that the SOC stock increased with the frequency of cultivation of grass ley. Positive effects of diversifying crop rotations on SOC, even if the total quantity of C input would not increase, is likely related to changes in the chemical composition of plant residues, soil microbial community, and cultivation operations (Cotrufo et al., 2013; Kallenbach et al., 2015; King & Blesh, 2018). However, increasing species diversity in annual rotations does not always induce detectable effects on SOC (King et al., 2020; Zuber et al., 2015). According to the most recent data, the cropping systems in Finland have not diversified (Peltonen‐Sainio et al., 2017), but during the last two decades, the area of grass ley has expanded (Statistics database, 2022) as has happened also in neighboring country Sweden (Poeplau et al., 2015).

Approximate effects on SOC stock presented in Tables 1 and 2 show that over the relatively short observation period, the climate change has resulted in SOC losses with the similar magnitude or exceeding the beneficial effects of diversified or perennial grass‐dominated crop rotations. It is thus unlikely that even improved crop rotations are sufficient to counterbalance the climate change‐induced SOC losses, especially in the future climate projections.

The amount of manure‐derived C is likely to be higher in livestock than in plant production farms. Although manure is more recalcitrant to biodegradation and hence builds up SOC more efficiently than fresh plant residues (Kätterer et al., 2011), it is estimated that only about 12% of the total C input in arable soils derives from organic fertilizers including manure (Jacobs et al., 2020), which might explain the lack of discernible difference in SOC balance between the livestock and plant production farms of the present study.

Soil sampling in the present study was based on the fixed depth (FD) method, which is known to be sensitive to management‐induced variation in soil bulk density and tillage depth (Ellert & Bettany, 1995; Heikkinen et al., 2021; Wendt & Hauser, 2013). Ideally, soil sampling should be conducted in several layers following the calculation of SOC stock changes using the equivalent soil mass (ESM) method, which defines soil layers based on the mineral soil mass. However, as discussed above the management‐related results presented in this study are realistic with respect to both magnitude and direction. The sampling method is assumed to have no impact on the findings associated with the climate change and SOC content trends.

### 4.3 Role of historical land use

Soils with a high initial SOC‐to‐fine‐fraction ratio were found prone to lose SOC (Tables 1 and 2), which is explained by the main role of the silt and clay fraction in the protection and stabilization of SOC (Baldock & Skjemstad, 2000; Kögel‐Knabner et al., 2008; Six et al., 2002). The formation of organo‐mineral complexes and incorporation of SOC into soil aggregates defines the maximum SOC storage capacity. Above the saturation level the SOC is in the form of particulate organic matter, which is expected to be more vulnerable to decomposition (Cotrufo et al., 2019; King et al., 2019; Matus, 2021; Poeplau et al., 2018; Stewart et al., 2008). The high SOC‐to‐fine‐fraction ratio of the bulk soil thus indicates that a larger share of the SOC is susceptible to decomposition than in low SOC‐to‐fine‐fraction ratio soils with available stabilization capacity. Soil survey‐based study by Bellamy et al. (2005) showed that the relative rates of SOC loss tended to increase with an increase in the initial SOC content, whereas soils low in SOC appeared to gain SOC.

The present study showed that the observed SOC‐to‐fine‐fraction ratio was not only influenced by the past land use type from which agricultural land was converted but also by the duration of cultivation. In plots situated in fields with >100 years history of cultivation the SOC‐to‐fine‐fraction ratio was approximately 0.03–0.05 and the changes in SOC content had leveled out. These soils are apparently reaching a steady state in relation to the properties and management of the soil.

In contrast, former organic soils (OM > 20%), that were most likely established on water‐logged peatlands were found to have high SOC‐to‐fine‐fraction ratios (typically 0.06–0.18) and the SOC loss was considerably faster than on average. Boreal regions are characterized by an abundance of peatland soils (Xu et al., 2018), and large areas of these soils have been drained for crop cultivation. For instance, in Finland cultivated organic soils cover approximately 14% of the agricultural land area (Kekkonen et al., 2019). As the C‐rich soil layer of these former peatlands gradually thins out due to the decomposition of organic matter, the mineral subsoil gets mixed with the organic topsoil finally turning the soil into mineral soil (OM < 20% as defined in this study) with a high SOC‐to‐fine‐fraction ratio. High C emissions related to the cultivation of peatland soils are well established (Kasimir‐Klemedtsson et al., 1997; Qiu et al., 2021), but the present study also evidences that these soils are vulnerable to SOC loss still long after turning into mineral soils. These findings are in agreement with the study by Smith et al. (2007) showing that the SOC loss observed in England and Wales (Bellamy et al., 2005) is most likely attributed mainly to drainage and past land use particularly in soils rich in SOC.

### 4.4 Bayesian modeling of SOC change

SOC measurements are characterized by high uncertainty due to high spatial variation of the SOC and errors related to sampling and analysis (Goidts et al., 2009). Although in this study sampling plots were located using GPS, sampling was done by trained field technicians, and SOC contents were determined in an accomplished research laboratory, some observed changes in SOC contents between 2009–2018 were up to one order of magnitude greater than what can be considered realistic (Figure 2). The specific shape of the SOC distribution and the extreme outliers pose a challenge for modeling. However, the Bayesian mixing‐model approach with two distributions not only manages to reproduce the observed distribution extremely well (Supplement 2) but can also be justified by the sampling procedure, since some of the plots are likely not to be relocated accurately enough and due to small‐scale within plot variability in SOC (Poeplau et al., 2022). Thus, we would expect some fraction of the observations to be roughly normally distributed with variance approximately related to the measurement accuracy and the rest of the points to follow another distribution with much greater variance. The Bayesian approach used here has an additional benefit that future measurements can be easily combined with the present ones. While such combining could be done using 2009–2018 and 1998–2009 datasets, the lower spatial accuracy of 1998–2009 data makes it practically obsolete in this case.

### 4.5 Concluding remarks

In this study, we demonstrated that the observed changes in SOC content in boreal agricultural land result from combined effects of climate change, cultivation practices, and the historical land use, that influence SOC on different timescales. There is potential to mitigate climate change by improved management of boreal agricultural soils, but under the future‐climate projections, there is a notable risk that climate change‐related losses of SOC to the atmosphere hamper the reaching of such goals. Despite the relatively small area of boreal agricultural soils, the results of the present study are alarming, especially if they indicate that climate‐driven SOC losses also occur in other boreal ecosystems, such as forest and wetlands with considerably larger land areas and SOC stocks. The results emphasize the importance of the reduction of greenhouse gas emissions to avoid the highly unpredictable effects of climate change‐induced SOC losses on the acceleration of planetary warming.

## CONFLICT OF INTEREST

The authors declare no competing interests.

## AUTHOR CONTRIBUTIONS

J.H. conceptualized the study and prepared the data. J.K. performed the statistical analysis. All authors contributed to the interpretation of data and drafting the manuscript.

## Supporting information

Supplementary Material

Supplementary Material

## Data Availability

The data that support the findings of this study are available on request from the corresponding author. The data are not publicly available due to privacy or ethical restrictions. Official register on cropping system and farm type which was used in the sampling site classification can be requested from Finnish Food Authority: https://www.ruokavirasto.fi/tietoa‐meista/avointieto/tiedonluovutukset/.
