# Supplementary material for: Climate change induces carbon loss of arable mineral soils in boreal conditions
Source: Glob Chang Biol. 2022 Apr 1;28(12):3960–73. doi: 10.1111/gcb.16164 (PMC9325001; doi:10.1111/gcb.16164)
Supplement: Supplementary file 1 — Supplementary Material [file GCB-28-3960-s002.docx]

**Supplement 1: Analysis of 1998 and 2009 dataset**


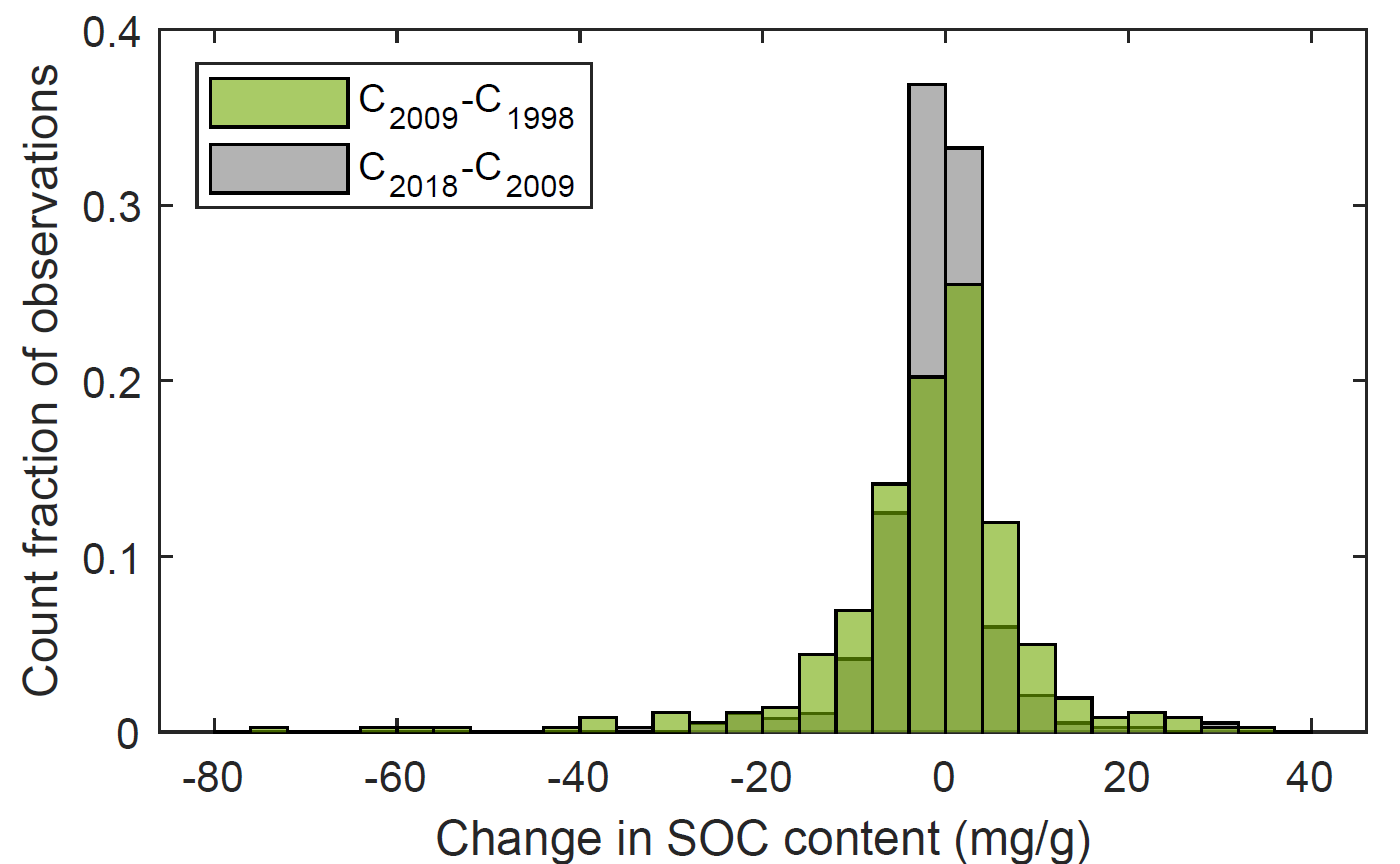


**Figure 1**. Count fraction of observed changes in SOC content. In comparison to the 2009-2018 (n=385) measured changes in SOC content the variance of the 1998-2009 (n=361) data is considerably higher due to lack of GPS based positioning of the sampling plots.


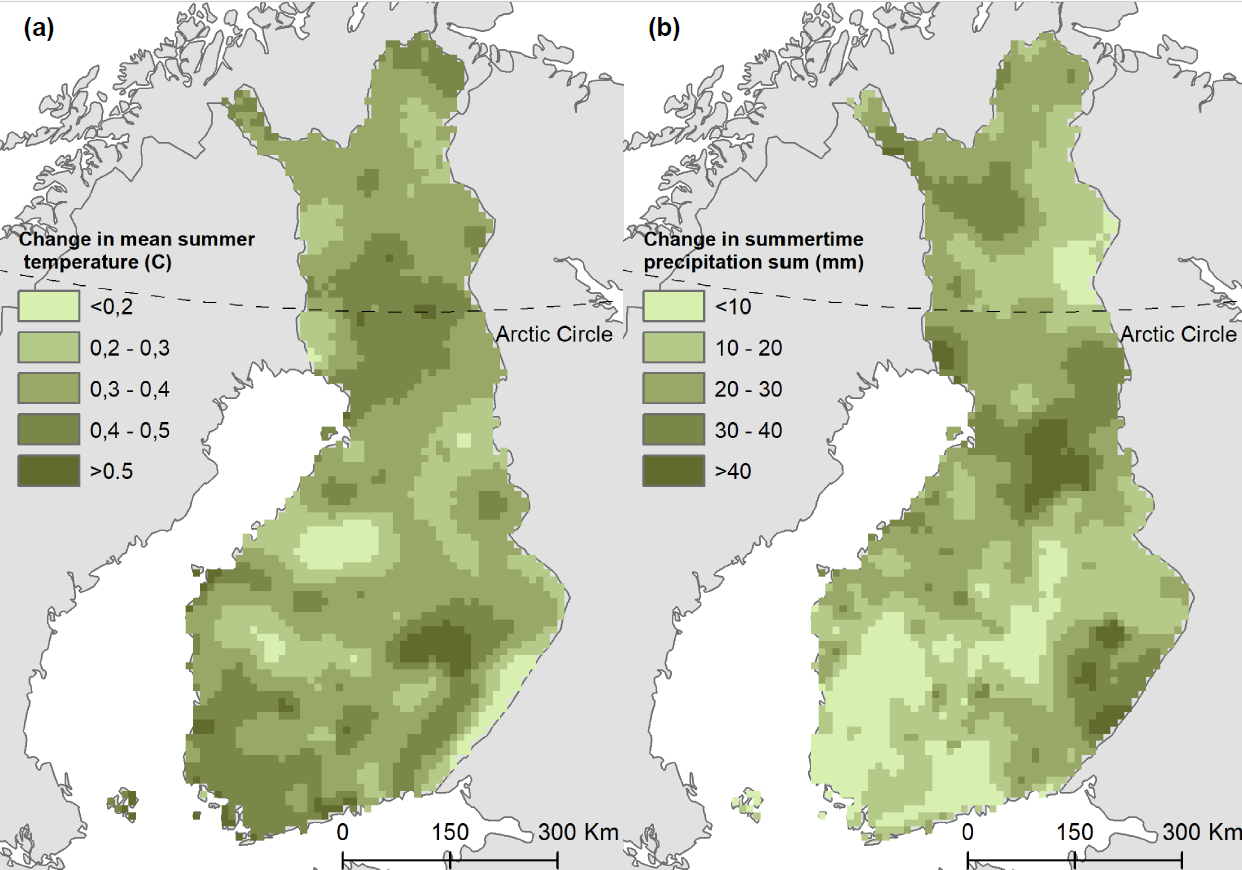


**Figure 2.** The change in summertime mean temperature (a) and precipitation sum (b) between 1998 and 2009 in 10 km x 10 km grid according to climate grid by Finnish Meteorological Institute.


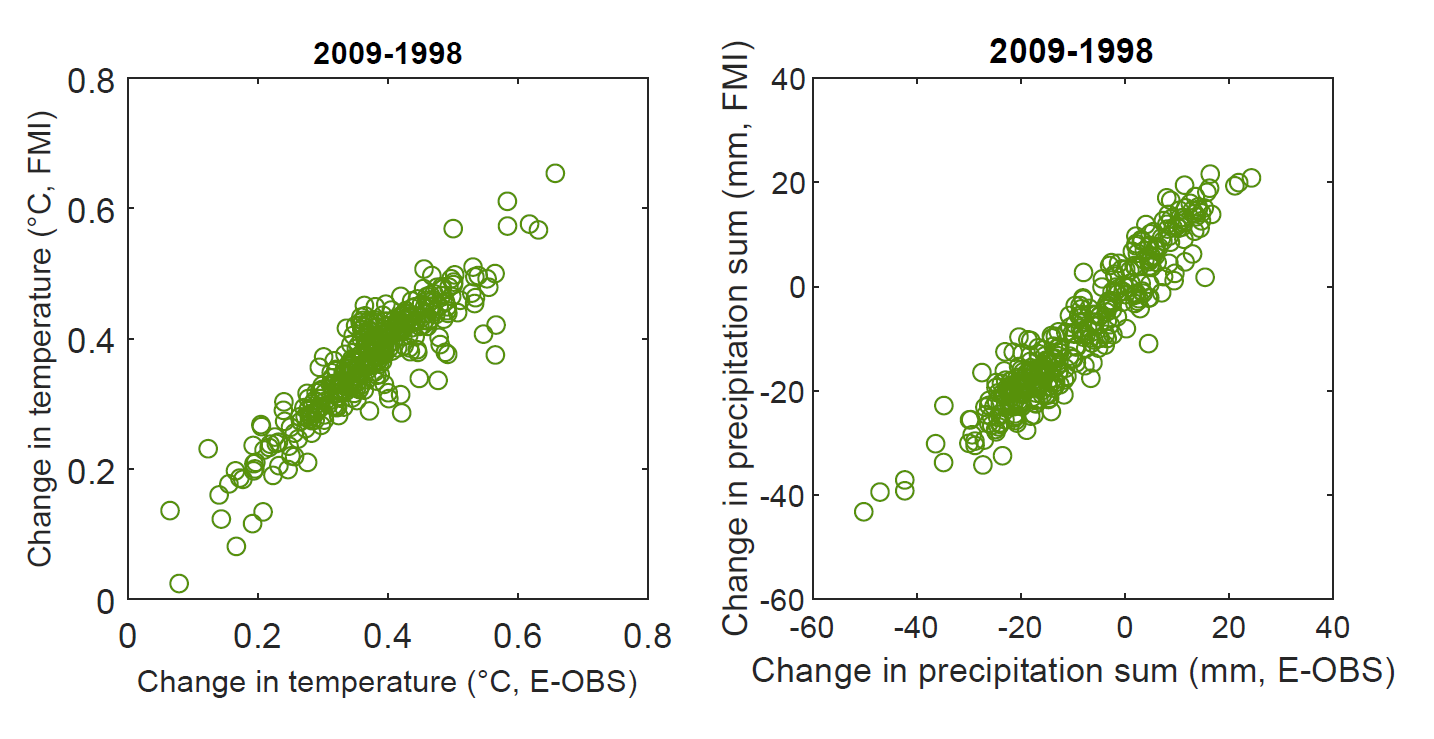


**Figure 3.** Comparison of change in summer (May-Sep) temperature and precipitation sum obtained from Finnish Meteorological Institute (FMI) and E-OBS climate grids.

**Table 1**. Results of the modelling of 1998-2009 data. Effect of climate change, management practices, and SOC-to-fine-fraction ratio on SOC content change $(E\left( \theta| y \right)$) using E-OBS and FMI climate data, 80 % (equally tailed) probability interval, and probability for positive effect (P($\theta$>0),%).

|  |  | **E-OBS** |  |  | **FMI** |  |  |
| --- | --- | --- | --- | --- | --- | --- | --- |
|  | $\mathbf{E}\boldsymbol{(\theta\vert y)}$ | $\boldsymbol{80 \%}\mathbf{P.I.}$ | **P(**$\boldsymbol{\theta}$**>0)** | $\mathbf{E}\boldsymbol{(\theta\vert y)}$ | $\boldsymbol{80 \%}\mathbf{P.I.}$ | **P(**$\boldsymbol{\theta}$**>0)** |  |
| $\boldsymbol{\beta}_{\mathbf{change P}}$ | -0.001 | (-0.005,0.003) | 33 | 0.000 | (-0.003,0.004) | 57 | |
| $\boldsymbol{\beta}_{\mathbf{change T}}$ | 0.112 | (-0.328,0.573) | 63 | 0.106 | (-0.380,0.594) | 61 | |
| $\boldsymbol{\beta}_{\mathbf{animal}}$ | -0.084 | (-0.176,0.009) | 12 | -0.090 | (-0.182,0.003) | 11 | |
| $\boldsymbol{\beta}_{\mathbf{diverse}}$ | 0.078 | (-0.050,0.205) | 78 | 0.081 | (-0.048,0.210) | 79 | |
| $\boldsymbol{\beta}_{\mathbf{perennial}}$ | 0.109 | (-0.007,0.226) | 89 | 0.102 | (-0.015,0.218) | 87 | |
| $\boldsymbol{\beta}_{\mathbf{rotation}}$ | 0.249 | (0.130,0.369) | 99.6 | 0.244 | (0.125,0.364) | 99.6 | |
| $\boldsymbol{\beta}_{\mathbf{log(OrgC/fine)}}$ | -0.340 | (-0.403,-0.278) | <0.1 | -0.346 | (-0.408,-0.285) | <0.1 | |
| $\boldsymbol{\mu}$ | -0.230 | (-0.428,-0.034) | 7 | -0.202 | (-0.406,0.001) | 10 | |
| $\boldsymbol{\pi}$ | 0.79 | (0.73.0.84) | 100 | 0.79 | (0.73.0.84) | 100 | |
| $\boldsymbol{1-}\boldsymbol{\pi}$ | 0.21 | (0.16.0.27) | 100 | 0.21 | (0.16.0.27) | 100 | |
| $\boldsymbol{\sigma}_{\boldsymbol{1}}$ | 0.475 | (0.434,0.516) | 100 | 0.475 | (0.433,0.516) | 100 | |
| $\boldsymbol{\sigma}_{\boldsymbol{3}}$ | 1.973 | (1.745,2.209) | 100 | 1.973 | (1.736,2.209) | 100 | |
| $\boldsymbol{\sigma}_{\mathbf{group}}$ | 0.085 | (0.018,0.160) | 100 | 0.085 | (0.018,0.159) | 100 | |


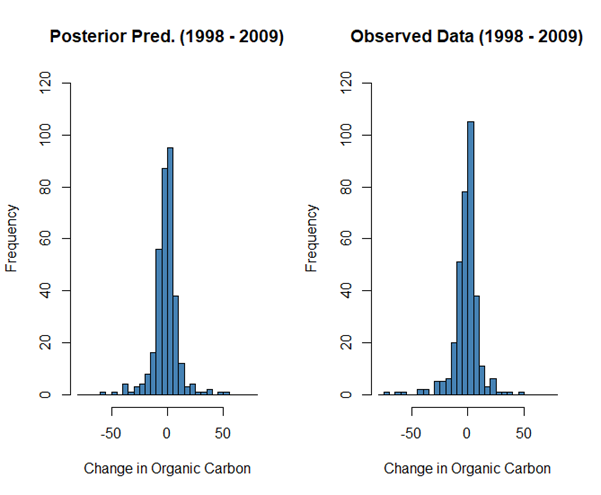


**Figure 4.** Simulated sample (with same n=361 as the data) from posterior predictive distribution and the observed data.
