# Supplementary material for: Climate change induces carbon loss of arable mineral soils in boreal conditions
Source: Glob Chang Biol. 2022 Apr 1;28(12):3960–73. doi: 10.1111/gcb.16164 (PMC9325001; doi:10.1111/gcb.16164)
Supplement: Supplementary file 2 — Supplementary Material [file GCB-28-3960-s001.docx]

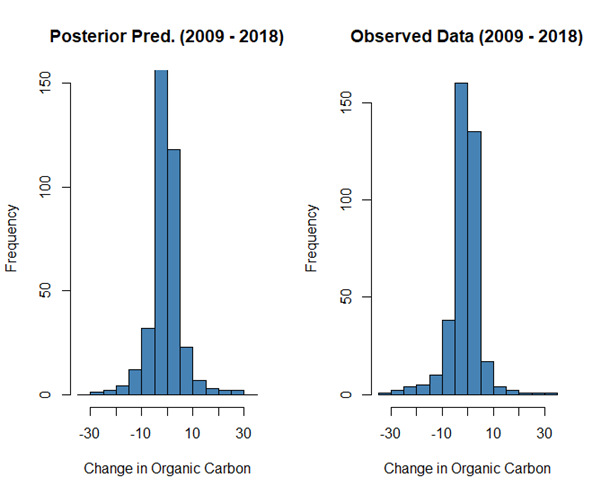


**Figure 1**. Simulated sample from posterior predictive distribution and the observed data.


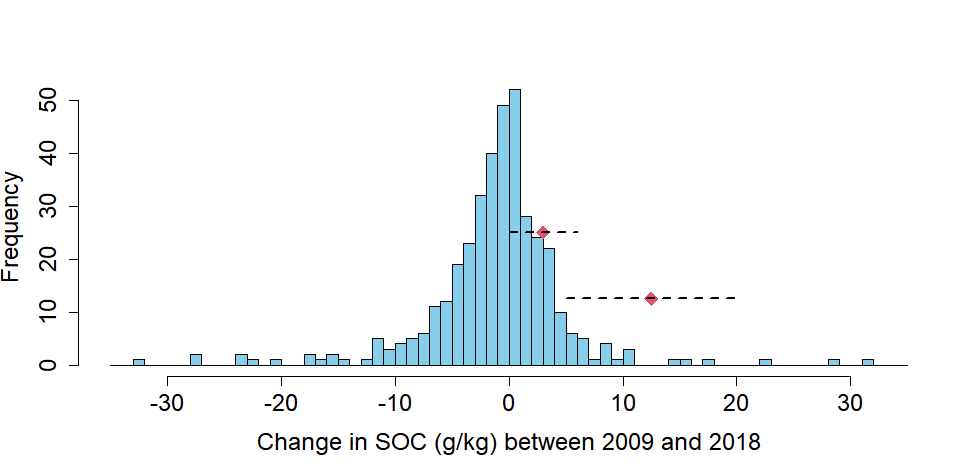


**Figure 2**. Graphical investigation for selecting the weak priors for the standard deviations. The standard deviation of the whole distribution is 6.1, and the narrow normal distribution thus has a standard deviation of roughly 3 which is why a uniform distribution Unif(0.01,6) is a conservative choice. This was also used as the prior for the standard deviation of the measurement group effect. The values vary between –35 and 35, and with roughly 10-30% of the distribution belonging to this wider distribution, we expect 35 to be somewhere around 2 to 4 standard deviations. Adopting a prior distribution Unif(5,20) for the difference between the narrower and the wider distributions is thus a reasonable limitation.
